# Natural history of groin hernias in women and factors leading to delay in repair: a single-institution study

**DOI:** 10.1007/s00464-025-11709-1

**Published:** 2025-04-15

**Authors:** Nathan C. English, Caleb Hood, Britney Corey, Abhishek D. Parmar

**Affiliations:** 1 Division of Gastrointestinal Surgery, Department of Surgery, University of Alabama at Birmingham, 1808 7th Avenue South, Boshell Diabetes Building #525, Birmingham, AL 35233 USA; 2 Department of Surgery, University of Cape Town, Cape Town, South Africa

**Keywords:** Female, Groin hernia, Natural history

## Abstract

**Background:**

The objective of this study was to describe the natural history of groin hernias in women at a high-volume tertiary medical center. Specifically, we abstracted the duration of symptoms prior to diagnosis, imaging modalities used, and operative findings. We hypothesized that females would experience a protracted preoperative clinical course.

**Methods:**

Our institutional hernia database was queried for elective groin hernia repairs from January 2018 to July 2023. Analyses were used to measure and describe demographics, clinical characteristics, and operative findings. In addition, patients’ zip codes were linked to census track area deprivation index (ADI) values and a semi-qualitative inquiry was performed to explore reasons for the protracted preoperative clinical course.

**Results:**

Among 1331 patients, 8.4% were female. Majority were Caucasian (68.8%) and overweight (BMI 27.3 ± 5.8), averaging 61.2 years of age. Majority reported non-specific groin pain (73.8%) and an intermittent groin bulge (48.8%), with 40% experiencing symptoms for > 1 year. Patients averaged 1.2 clinic visits before seeing a surgeon. Indirect inguinal hernias were the most common (81.3%), followed by femoral (35%) and direct (26.3%). Sixty-three patients had preoperative imaging, including CT (56.8%), US (39.2%), and MRI (4.0%). The most common surgical approach was robotic (68.8%) followed by laparoscopic-TEP (22.5%). When stratified by duration of symptoms, ADI did not differ among our cohort (p = 0.497). Patient-related reasons for delaying surgery included interpersonal stressors (3.1%), symptoms not limiting ADLs (34.4%), and fear of mesh complications (3.1%). Providers advised against surgery due to malnutrition (3.1%), multiple prior repairs (9.4%), concomitant infection (3.1%), and severe ascites (6.3%).

**Conclusion:**

Our study provides some insight into reasons for delay in inguinal hernia repair for women. While many reported symptoms for over a year, a minority sought treatment until they were ready to proceed with surgery. Future qualitative studies are needed to more thoroughly assess female’s perspectives with groin hernias.

Groin hernias are a common surgical problem, with over 20 million patients undergoing surgical repair annually worldwide [1]. Of these, 750,000 are performed in the USA [2]. Compared to males, who have a 27–43% lifetime risk for developing a groin hernia, females have only a 3–6% lifetime risk of developing a groin hernia [3]. As a result, females account for only 10% of all patients undergoing herniorrhaphy [4]. In addition, the clinical signs and symptoms of groin hernias are variable and may be quite subtle in females [5]. These include non-specific groin pain, paraesthesia in a specific dermatomal distribution, or more acute symptoms of an irreducible bulge with or without features of intestinal obstruction [6]. Some patients may also experience symptoms intermittently, becoming manifest only when in certain positions (upright > supine) or during episodes of raised intra-abdominal pressure (straining, coughing, sneezing) [7]. Furthermore, up to one-third of patients do not volunteer symptoms directly attributable to a hernia [6]. As a result, female patients often present to their family practitioner or non-surgical healthcare provider [8], gynecologist [9], or even psychiatrists prior to seeing a surgeon. Consequently, vague groin symptoms may be attributed to other more common pathology, including osteo-arthritis of the pelvic girdle, gastroenteritis, lymphadenopathy, diverticulitis, or constipation, compounded by equivocal groin examination findings [10, 11]. Ultimately, this may lead to a delay in care for women which exposes them to a risk for emergency complications.

In addition, a knowledge gap remains of factors that lead to delay in hernia repair for women. There is a significant sex bias in surgical research [12] and this is pronounced for studies on females with groin hernias [13]. For men, there are several seminal articles establishing the natural history of inguinal hernias [14, 15]. Fitzgibbons et al. clearly delineated the low relative risk for hernia complications in men managed by watchful waiting in a well-designed randomized controlled trial [16]. In this study, men with minimally symptomatic inguinal hernias were found to have a low risk for progression to incarceration or strangulation over five years, supporting a strategy of watchful waiting for men [16]. However, for women, no such standards can exist, given the dearth of clinical evidence supporting the watchful waiting approach in this population.

This multitude of factors significantly complicate the care of women with groin hernias, leading toward a protracted preoperative clinical course for these patients. Prior reports have noted a 3–4-fold higher rate of emergency hernia repairs in women compared to men, which may be due to this delay in care [17]. The objective of this study was to first describe our experience with the natural history of groin hernias in women at our institution, a tertiary care academic center with a high-volume experience with inguinal hernia repair. Specifically, we aimed to elucidate personal, demographic, and socioeconomic factors that may have contributed to the protracted preoperative clinical course for these patients. In addition, we assessed preoperative clinical characteristics and operative findings at elective surgical repair.

## Material and methods

### Study design, inclusion, and exclusion criteria

**Fig. 1:**
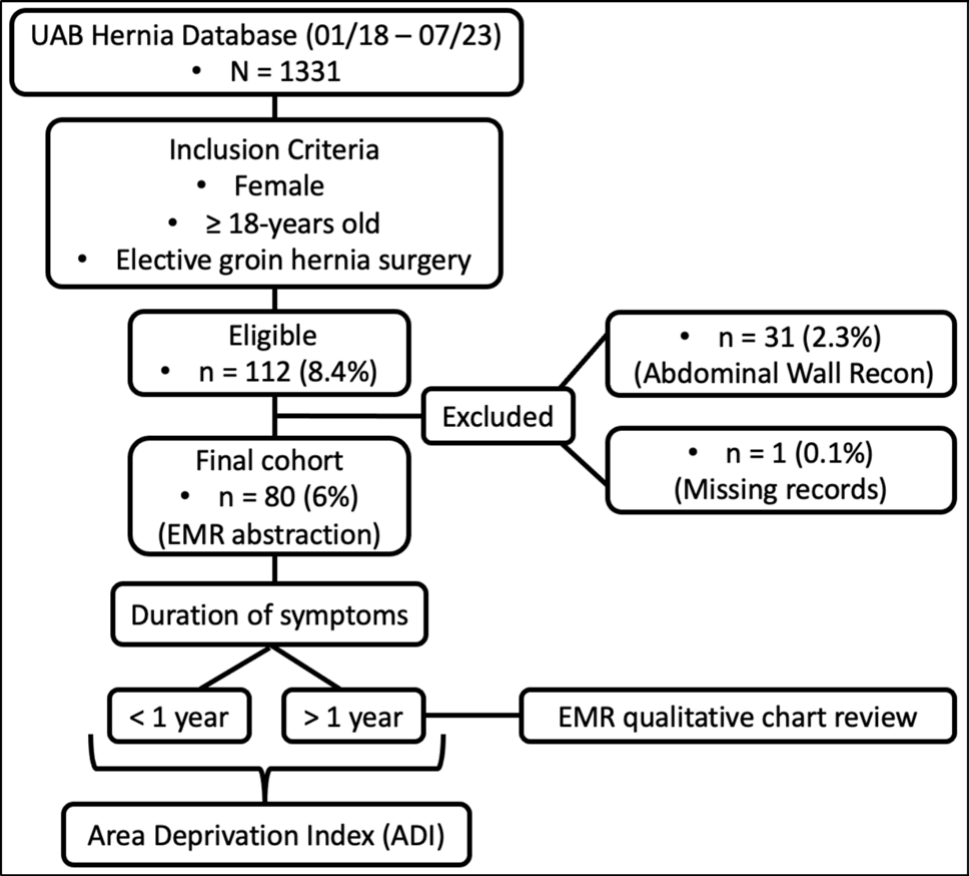
Inclusion and exclusion criteria

This study was a retrospective cohort analysis of a prospectively maintained hernia database at the University of Alabama at Birmingham (UAB) Medical Center, Division of Gastrointestinal Surgery, and affiliated hospitals from January 2018 to July 2023. The UAB hernia database was filtered using Current Procedural Terminology (CPT) codes to capture all patients who had undergone elective surgery for direct inguinal, indirect inguinal, and femoral hernias during the study period. The index search revealed a total of 1331 patients. We excluded males (n = 1219), patients who had incidental groin hernias repaired at the time of abdominal wall reconstruction (AWR) (n = 31), and those with incomplete records (n = 1). The final cohort comprised 80 adult female patients (≥ 18 years old) who underwent herniorrhaphy for clinically detectable groin pathology in the preoperative period (Fig. 1). The study protocol was part of an approved protocol by the UAB Institutional Review Board (IRB) (IRB-300003313). The Strengthening the Reporting of Observational studies in Epidemiology (STROBE) guidelines was used for study reporting [18].

### Data collection

The CPT codes related to the procedures of the 80 female patients included in the study were linked to their medical record numbers (MRNs). Using the MRNs, we queried the institutional electronic medical records (EMR) to abstract basic demographics, preoperative characteristics, and intraoperative findings. In addition, the national percentile Area Deprivation Index (ADI) was abstracted from The Neighborhood Atlas® [19, 20] using the zip codes pertaining to patients’ most recent home address as documented in the EMR.

### Outcomes

The primary outcomes included the duration of symptoms prior to diagnosis, number of non-surgical physicians consulted, imaging modalities used in the preoperative period, and intraoperative findings. In order to achieve the study’s second objective, first we dichotomized patients into 2 groups according to the duration of symptoms (< 1 year vs. > 1 year) and assessed if differences exist across groups with respect to clinical factors, ADI and insurance status. ADI was selected as a validated composite measure of social/ neighborhood-level disadvantage that has been linked to access to care and surgical outcomes in prior reports [21, 22]. Similarly, for the purposes of this study, ADI proxied the ability to access care [23] and was categorized into quartiles (First: 0–25; Second: 26–49; Third: 50–75; and Fourth: > 75) for analysis. Second, we performed an extensive EMR search backdating three years prior to surgical repair for the group of patients with symptoms > 1 year in order to elucidate any documented reason(s) for the protracted preoperative clinical course or delay in hernia repair.

### Statistical analysis

Descriptive statistics were conducted using frequencies and percentages for categorical variables, while continuous variables were summarized with means ± standard deviation and/or medians with minimum and maximum values. Bivariate comparison of all variables among patients stratified by duration of symptoms (< 1 year vs > 1 year) was performed using the Likelihood ratio Chi-square or Fisher’s exact test with a p < 0.05 considered statistically significant. The statistical analysis was performed using SAS software version 9.4 (SAS Institute, Cary NC).

## Results

Of the 1331 patients identified in the database who underwent elective inguinal hernia repair during the five-year study period, 112 (8.4%) met the initial inclusion criteria. Thirty-one patients (2.3%) who had incidental groin hernias repaired at the time of abdominal wall reconstruction and 1 (0.1%) with incomplete records were excluded. The final cohort comprised of 80 (6%) female patients. The mean age of participants was 61.2 years (± 15.4, range 23–92), and majority were Caucasian (68.8%), and non-smokers (92.5%) (Table 1).

**Table 1:** Demographics (N = 80)

Variable	n (%)
Age (years)	
Mean ± SD	61 ± 15
Minimum	23
Maximum	92
Race	
White	55 (68.8)
Black	20 (25.0)
Unknown	2 (2.5)
Hispanic/Latino	1 (1.3)
Non-HispanicLlatino	1 (1.3)
Asian	1 (1.3)
Insurance status	
Medicare	35 (43.7)
Private	25 (32.2)
Viva	12 (15.0)
Medicaid	3 (3.7)
Charity	3 (3.7)
Unknown	2 (2.5)
Employment status	
Retired	32 (40.0)
Employed	28 (35.0)
Unemployed	10 (12.5)
Unknown	6 (7.5)
Social grant	4 (5.0)
Smoker	
No	74 (92.5)
Yes	6 (7.5)

### Preoperative characteristics

**Fig. 2:**
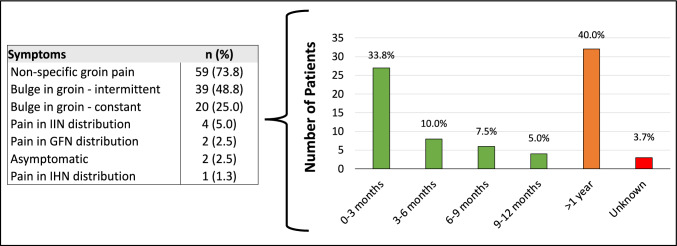
Duration of symptoms (N = 80)

The majority of patients were overweight with a mean BMI of 27.3 (± 5.8, range 16.4 – 48.9) and were majority ASA III (67.5%). The most common symptoms encountered included non-specific groin pain (73.8%) and an intermittent groin bulge (48.8%). Many patients (40%) experienced symptoms for > 1 year, while 33.8% experienced symptoms for less than 3 months (Fig. 2). Patients averaged 1.2 clinic visits regarding their hernia and waited an average of 22.6 (range 1–90) days prior to seeing a surgeon. While majority (60%) were in-network referrals, there was no significant difference with respect to the duration of symptoms prior to surgical repair when compared with those referred out-of-network or self-referrals, respectively (p = 0.934). Sixty-three patients (78.8%) had preoperative imaging. The most common modality was Computed Tomography (CT) (56.8%), followed by Ultrasound (US) (39.2%) and Magnetic Resonance Imaging (MRI) (4.0%) (Table 2).

**Table 2:** Preoperative characteristics (N = 80)

Variable	n (%)
BMI	
Mean ± SD	27.3 ± 5.8
Minimum	16.4
Maximum	48.9
ASA	
I	4 (5.0)
II	22 (27.5)
III	54 (67.5)
Comorbidities	
Other	68 (85.0)
Hypertension (HPT)	38 (48.10)
Diabetes (DMII)	9 (11.3)
None	7 (8.8)
COPD	6 (7.5)
Symptoms	
Non-specific groin pain	59 (73.8)
Bulge in groin—intermittent	39 (48.8)
Bulge in groin—constant	20 (25.0)
Pain in IIN distribution	4 (5.0)
Pain in GFN distribution	2 (2.5)
Asymptomatic	2 (2.5)
Pain in IHN distribution	1 (1.3)
Duration of symptoms	
0–3 months	27 (33.8)
3–6 months	8 (10.0)
6–9 months	6 (7.5)
9–12 months	4 (5.0)
> 1 year	32 (40.0)
Unknown	3 (3.7)
Non-surgeon physician consulted	
Yes	64 (80.0)
No	16 (20.0)
No. of non-surgical physicians consulted	
Mean ± SD	0.89 ± 0.53
Minimum	0
Maximum	2
Non-surgical physician consulted	
Yes	
Other (primary care physician)	53 (66.3)
Gynecologist	10 (12.5)
Psychiatrist	1 (1.2)
No	16 (20.0)
Referral type	
In-network	60 (75)
Out-of-network	11 (13.7)
Self-referral	9 (11.3)
Days from referral to first clinic visit	
Mean ± SD	22.6 ± 19.8
Minimum	1
Maximum	90
Hernia occurrence	
Index	70 (87.5)
Recurrent	9 (11.3)
Index/Recurrence*	1 (1.2)
Laterality	
Unilateral	67 (83.8)
Bilateral	13 (16.2)
Type of defect	
Defect—unspecified	52 (65.0)
Inguinal—indirect	12 (15.0)
No defect noted	10 (12.5)
Femoral	6 (7.5)
Inguinal—direct	2 (2.5)
Preoperative imaging	
Yes	63 (78.8)
No	17 (21.2)
Image modality	
CT	42 (56.8)
US	29 (39.2)
MRI	3 (4.0)

*BMI* body mass index, *SD* standard deviation, *ASA* American Society of Anesthesiologists, *CT* computed tomography scan, *US* ultrasound scan, *MRI* magnetic resonance image

^*^This patient had an index hernia on one side and a recurrence on the contralateral side

### Operative findings

**Fig. 3:**
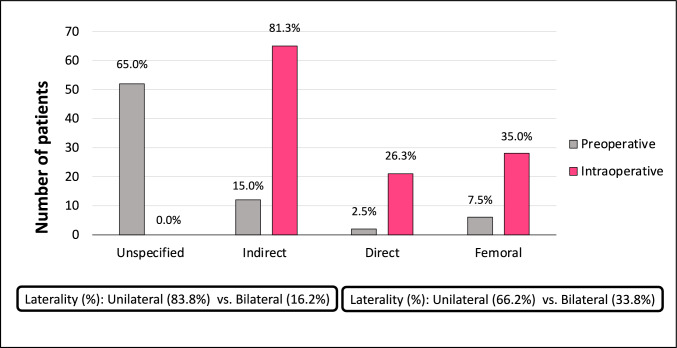
Hernia defect preoperative vs. intraoperative (N = 80)

The robotic platform was the most common surgical approach used (68.8%), followed by laparoscopic-TEP (22.5%) (Table 3). While only 13 patients (16.3%) were noted to have bilateral hernias in the preoperative period, a total of 27 (33.8%) were noted intraoperatively. Indirect inguinal hernias were the most common type of hernia identified (81.3%), followed by femoral (35%) and direct (26.3%) (Fig. 3).

**Table 3:** Operative findings and outcomes (N = 80)

Variable	n (%)
Approach	
Robotic	55 (68.7)
Laparoscopic—TEP	18 (22.5)
Laparoscopic—TAPP	5 (6.2)
Conversion to open	1 (1.3)
Open	1 (1.3)
Laterality	
Unilateral	53 (66.2)
Bilateral	27 (33.8)
Type of defect (Intraoperative)	
Inguinal—indirect	65 (81.3)
Femoral	28 (35.0)
Inguinal—direct	21 (26.3)
Operative complications	
Vascular injury*	2 (2.5)
Nerve injury	0 (0.0)
Bowel injury	0 (0.0)
30-day outcome	
Discharged	79 (98.8)
Readmission	1 (1.2)

*TEP* totally extra-peritoneal, *TAPP* Trans-abdominal pre-peritoneal

*Injury noted to inferior epigastric artery

### Factors influencing delay in surgical care

**Fig. 4:**
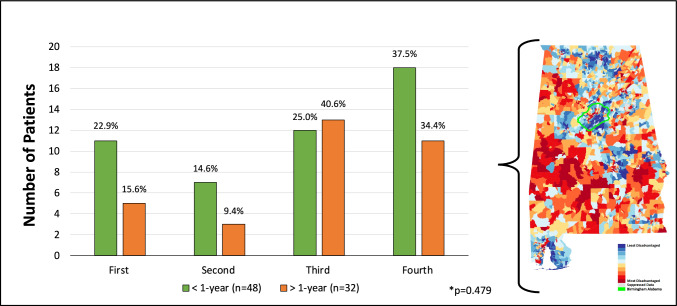
Area Deprivation Index (ADI) quartiles by duration of symptoms* (N = 80). Note: heatmap of Alabama summarizing census track ADI, adapted from: University of Wisconsin School of Medicine and Public Health. Area Deprivation Index. https://www.neighborhoodatlas.medicine.wisc.edu/mapping. Published 2021

**Fig. 5:**
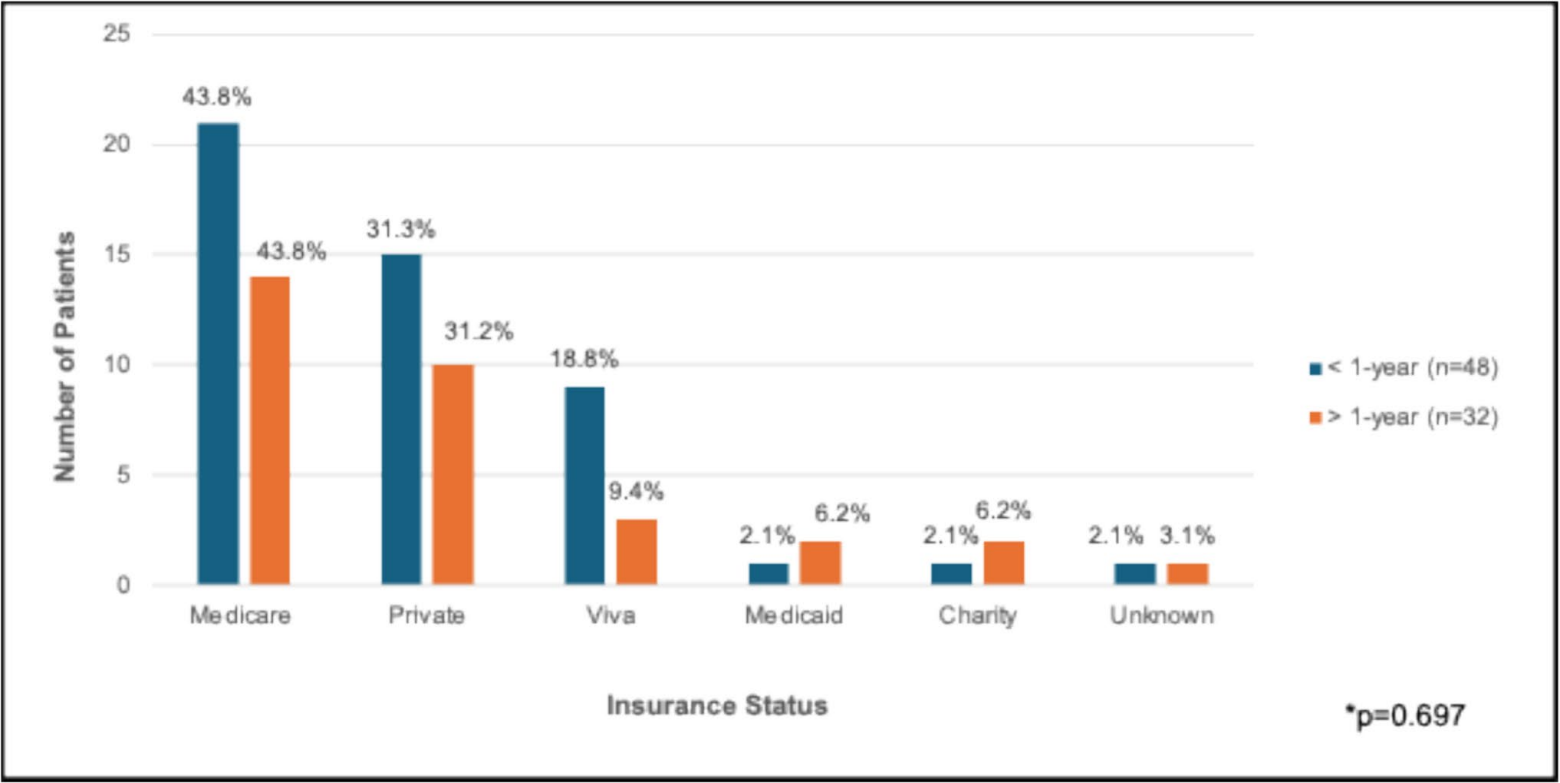
Insurance status by duration of symptoms* (N = 80)

The ADI and insurance status did not differ significantly among our cohort when stratified by duration of symptoms (p = 0.479 and p = 0.697, respectively) (Fig. 4 and 5). Possible reasons for the delay in surgical repair among patients who experienced symptoms for > 1 year as abstracted from the EMR were classified into patient- and provider-related factors (Table 4). Reasons patients previously declined surgery or referral to a surgeon included (1) experiencing significant life stressors at the time of initial consultation (i.e., family member with a terminal illness), (2) symptoms not interfering with activities of daily living (ADLs), (3) fears surrounding mesh related complications, (4) requesting to discuss with family prior to operative repair, and (5) a fear of surgical evaluation of groin swelling. Providers had previously advised against surgical repair due to (1) concerns of an adverse postoperative outcome in a patient with malnutrition, (2) uncertainty of benefit among patients who had undergone multiple previous repairs, (3) concomitant infection in a patient with recurrent urinary tract infections, (4) severe ascites in patients with cirrhosis, and (5) delay in care due to prior misdiagnoses.

**Table 4:** Factors influencing protracted (> 1 year) preoperative course (EMR abstraction) (N = 32)

Variable	n (%)
Patient factors—declining surgery	15 (46.9)
Multiple life stressors (husband with terminal illness)	1 (3.1)
Symptoms not interfering with ADLs	11 (34.4)
Fear of mesh complications	1 (3.1)
Patient wanted to discuss with family prior to surgery	1 (3.1)
Fear of surgical evaluation	1 (3.1)
Provider—advised against surgery	9 (28.1)
High risk due to low BMI (14.76)	1 (3.1)
Multiple previous repairs—unlikely to relieve symptoms	3 (9.4)
Concomitant infection (UTI)	1 (3.1)
Severe ascites	2 (6.3)
Misdiagnosed abdominal/ groin pain	
Unspecified post-partum abdominal pain	1 (3.1)
Abdominal pain attributed to previous uterine ablation	1 (3.1)
No exact reason noted	8 (25.0)

*ADLs* activities of daily living, *BMI* body mass index, *UTI* urinary tract infection

## Discussion

This study was a retrospective audit which evaluated the natural history of groin hernias among women at a high-volume tertiary medical center. Our study confirms prior findings in which females represent a small percentage (8.4%) of all groin hernia repairs performed [3]. We noted that a significant portion of our patients (40%, n = 32) experienced symptoms for longer than one year. Our study is rare in that we elicited specific reasons for this delay in treatment. Ultimately, the reasons for the delay in care were multifactorial and included patient centric as well as provider-driven factors. We did not identify disparities based on sociodemographic factors.

For patients experiencing delays in time to surgery, we found both patient and provider factors played a role. Surgeon-directed delays related to complexity or prohibitive risk of the operation played a smaller role compared to patient-driven practical reasons. While these factors were largely reasonable, 25% of our patients had no specific reason documented in the medical record, highlighting the need for future qualitative inquiry. Prior studies have found that minimal symptoms can be a driver for delay in seeking hernia repair [24]. McEntee et al. noted that even when symptomatic (strangulated hernia), some patients do not report to their primary care physician, while approximately one-third lack referral for surgical evaluation after consultation with non-surgical medical personnel [25]. In addition, of those who undergo surgical evaluation, high-risk comorbidities particularly among the elderly and limited social resources can often preclude timely repair [26]. As such, after noting the limited number of patients who progressed to elective hernia surgery following consultation in a preoperative optimization clinic, Ehlers et al. qualitatively assessed barriers to behavior change among a group of high-risk patients [27]. Nuanced patient-related barriers included a lack of agency for change (i.e., taking charge of goal-directed plans regarding food choices and engaging with weight loss resources offered by providers) and a lack of patient–provider concordance as some patients feared the risk of hernia-related emergencies while struggling to meet guidelines for elective repair. Furthermore, logistical barriers including a lack of transportation to access hospitals and clinics in addition to a lack of insurance were highlighted [27]. Our study mirrors these prior findings that practical logistics as well as anxiety can lead to delays in seeking surgical care.

In concert with these findings, prior reports assessing large datasets have demonstrated that uninsured and underinsured patients are less likely to undergo operative repair of their hernia compared to privately insured patients [28]. Compounding these disparities is the recent decline in Medicare reimbursement of hernia repairs [29] which is likely to portend additional surgical accessibility barriers for vulnerable patients. For our study population, we noted no significant differences with respect to ADI, a measure of healthcare access [23]. This suggests that at least for our healthcare system and catchment area that other factors may be influential, including the severity of symptoms, the impact surgery may have on patients’ immediate social and interpersonal relationships, and fears related to undergoing the surgery.

The lack of hernia awareness may play a role in the delay for women with groin hernias. A prior study by Kjaergaard et al. [30] has shown a dearth of physical exam for hernia in women to be relatively common, as approximately 40% of hernias causing mechanical bowel obstruction were missed owing to a lack of groin examination. These findings are further supported by observations from the Swedish Hernia Register assessing mortality due to femoral hernias, where it was noted that only 37.4% of patients had groin examinations before presentation to hospital [31]. Our study findings support this challenge, as only 81.3% of participants had a palpable defect on clinical examination, the majority of which were unspecified, i.e., the clinical defect was not attributed to a particular type of hernia. In addition, femoral hernia defects, which are more common in women, are generally smaller compared to inguinal hernias and with the rising incidence of obesity [32] compounding equivocal groin examination findings, it is increasingly difficult to distinguish it from an inguinal hernia [11]. Surgeons in the past have emphasized specific strategies to assess for occult hernias in women, including assessing for tenderness over the external ring[33], noting tenderness over the internal ring during Valsalva’s maneuver, and noting neuropathic pain in the distribution of the ilio-inguinal nerve (IIN) [34]. We did not identify the presence of neuropathic pain as a valuable strategy in our cohort, as only 8% experienced neuropathic pain, with 5% noted to be in the IIN distribution.

Our study also confirms prior findings highlighting the benefits of the minimally invasive approach in groin hernia repair for women, since synchronous femoral hernias are detected in up to 40% of cases and are otherwise frequently missed [35]. In our study, 97.5% of cases were performed via a minimally invasive approach, which revealed femoral and contralateral defects in 35.0% and 33.8% of cases, respectively. This is in contrast to the preoperative findings, where only 7.5% had a documented femoral hernia and 16.3% were noted to have bilateral defects.

Our study has several limitations. First, all patients were from a single institution, therefore, despite our institution serving a diverse population from both urban and rural settings [36], it may limit the generalizability of the results. Second, as patients’ most recent home address (zip code) was linked to the ADI at the time of data abstraction, we recognize that the results regarding sociodemographic factors may be biased as some may have relocated during the 5-year study period. Third, our cohort comprised entirely of patients who underwent elective hernia repairs, thereby limiting the applicability of the findings when considering all at-risk female patients with groin hernia. Finally, while our institutional EMR inquiry among those with symptoms for > 1 year yielded possible reasons for the delay in care in 75% of patients, we recognize that 25% had no specific reason documented. This knowledge gap should be addressed with future qualitative investigations among females with groin hernias.

## Conclusion

We have found that for our population of women with groin hernias, many will experience symptoms for over a year before seeking treatment. Patient anxieties regarding the operation and practical surgeon-directed delays played a role, emphasizing the imperative for earlier and more frequent referral to a surgeon. These may be addressed through educational efforts geared to primary care physicians and gynecologists about the safety of hernia repair. Much of the reasons for delay is still not known. Additionally, the incidence of non-palpable, occult contralateral, and femoral hernia defects were relatively high, supporting the application of minimally invasive techniques. Future qualitative studies are needed to more thoroughly assess the female experience with groin hernias.
